# Electrochemical and chemical dealloying of nanoporous anode materials for energy storage applications

**DOI:** 10.1080/14686996.2025.2451017

**Published:** 2025-01-31

**Authors:** Muhammad Afiq Irfan Mohd Shumiri, Abdillah Sani Mohd Najib, Andi Erwin Eka Putra, Nor Akmal Fadil

**Affiliations:** a Materials Research and Consultancy Group, Faculty of Mechanical Engineering, Universiti Teknologi Malaysia, Johor Bahru, Malaysia; b Department of Materials, Manufacturing and Industrial Engineering, Faculty of Mechanical Engineering, Universiti Teknologi Malaysia, Johor Bahru, Malaysia; c Battery and Advanced Materials Research Center, Hasanuddin University, Makassar, Indonesia

**Keywords:** Electrochemical dealloying, energy storage, porous structure, anode modifications, cycle stability, functionalized materials performance

## Abstract

Traditionally employed in alloy corrosion studies, dealloying has evolved into a versatile technique for fabricating advanced porous materials. The unique architecture of interconnected pore channels and continuous metal ligaments endows dealloyed materials with high surface-to-volume ratio, excellent electron conductivity, efficient mass transport and remarkable catalytic activity, positioning them at the forefront of nanomaterial applications with significant potential. However, reproducible synthesis of these structures remains challenging due to limitations in conventional dealloying techniques. Herein, this review attempts to consolidate recent progress in electrochemical and chemical dealloying methods for nanoporous anodes in energy storage and conversion applications. We begin by elucidating the fundamental mechanisms driving dealloying and evaluate key factors influencing dealloying conditions. Through a review of current research, we identify critical properties of dealloyed nanoporous anodes that warrant further investigation. Applications of these materials as anodes in metal-ion batteries, supercapacitors, water splitting and photocatalyst are discussed. Lastly, we address ongoing challenges in this field and propose perspectives on promising research directions. This review aims to inspire new pathways and foster the development of efficient dealloyed porous anodes for sustainable energy technologies.

## IMPACT STATEMENT

This review helps to consolidate recent advancements in electrochemical and chemical dealloying techniques for nanoporous anodes, highlighting critical properties, applications and challenges to inspire future research in sustainable energy technologies.

## GRAPHICAL ABSTRACT

**Figure:**
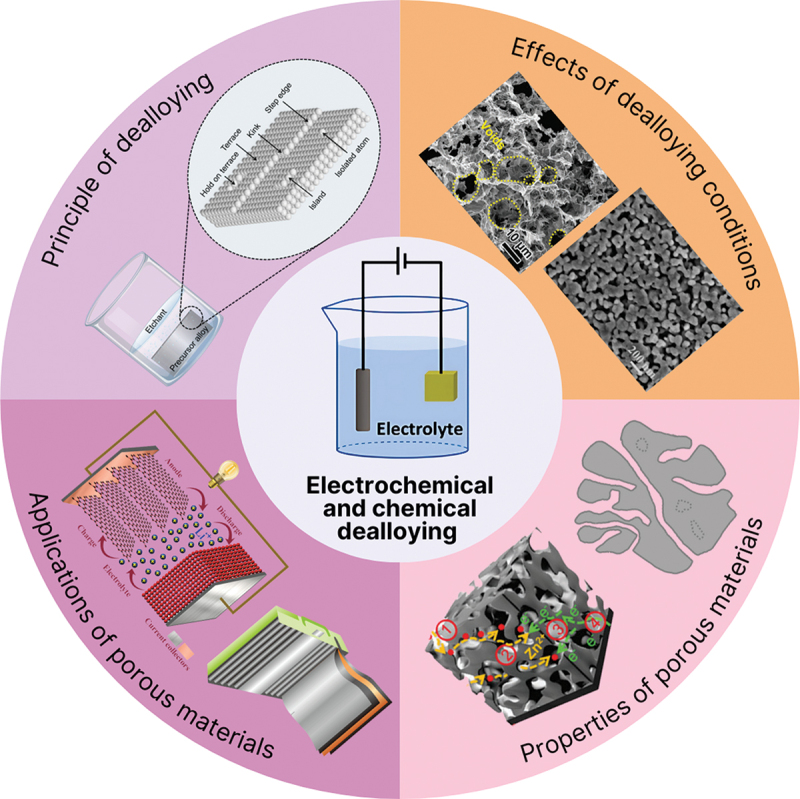

## Introduction

Historically, significant efforts have focused on preventing corrosion in alloys to avoid destructive degradation and extend material lifespan. However, corrosion processes have recently garnered renewed interest as a strategic approach to develop metallic nanoporous materials through dealloying. Electrochemical dealloying is a process initiated by applying external electric current which preferentially removes the more reactive component of precursor alloy. Apart from that, chemical dealloying occurs by spontaneous chemical reaction between the etchant and precursor alloy under free corrosion conditions. Both methods involve selective dissolution of the less noble element. However, while chemical dealloying is sometimes referred to as a purely chemical process, the mechanism is fundamentally electrochemical which driven by differences in chemical reactivity of the alloy components [1]. In both processes, the more reactive constituent dissolves and leaves behind porous structure of the more stable metal. One well-known example of material created by dealloying is Raney nickel. It has been widely used as catalyst in hydrogenation reactions [2]. Engineered by Murray Raney in 1927, Raney nickel has become a model to study on dealloying processes. Hitherto, the success has inspired further research into nanoporous metals and alloys for advanced applications including energy storage system (ESS) to reduce fossil fuel consumption.

High performance ESS play indispensable role across various sectors especially in transportation industry. For example, electric vehicles rely heavily on ESS for energy storage and delivery [3]. The effectiveness of these systems directly affects operational performance that enables key functions such as peak power output, energy capacity, cycle longevity and overall efficiency. Among the various types of ESS including batteries, supercapacitors and fuel cells, research has traditionally focused on cathode development, with considerable advancements in material design, structural refinement and surface modification [4]. However, optimizing the anode components remain significant challenge. Dealloyed nanoporous anodes have shown great potential to enhance the performance of ESS, but their full capabilities are yet to be fully explored. Encouragingly, recent advancements in electrochemical dealloying techniques have created new opportunities to improve the design of porous materials. A comprehensive review of these developments and analysis of the fundamental principles could pave the way for more efficient optimization of nanoporous anodes. These efforts could provide valuable insights and strategies to further improve energy storage applications.

The growing interest in dealloyed nanoporous anodes for energy storage is largely due to their unique microstructures. The surface structure consists of continuous metal ligaments and interconnected pore channels which provide many advantages. The high surface area-to-volume ratio improves catalytic charge transfer processes [5]. The continuous ligaments facilitate fast electron conduction, while the open-pore channels enable efficient mass transport. Together, these features make dealloyed nanoporous anodes highly suitable for electrochemical energy conversion and storage applications. Importantly, the porosity can be finely tuned by adjusting various dealloying parameters [6]. To the best of our knowledge, the commonly adopted dealloying conditions are often limited. Undoubtedly, factors such as temperature, concentration of the leaching solution, dealloying time, potential, precursor master alloy composition, type and phase distribution influence the final metal-and-void structure. By carefully controlling these parameters, researchers can create nanoporous materials tailored to the specific requirements of energy storage devices including the creation of hierarchical pore structures beyond simple unimodal designs [7]. For this reason, there is a need to systematically discuss on controllable dealloying methods for synthesizing nanoporous anode.

Herein, in this review, we explore the potential of electrochemically dealloyed nanoporous anodes for energy storage applications. We begin by describing the fundamental principles of dealloying to provide clear understanding of the process. Next, we discuss the effects of various dealloying parameters on the porosity by highlighting the relationship between processing conditions and material performance. Following this, we explore the distinctive characteristics of dealloyed nanoporous anodes that make them suitable for functional applications. We review recent research on the adoption of dealloyed nanoporous materials in lithium-ion batteries (LIBs), zinc-ion batteries (ZIBs), sodium-ion batteries (SIBs), potassium-ion batteries (KIBs), supercapacitors, anodic oxygen evolution reaction (OER) of water splitting and photocatalyst applications. In the final section, we address existing challenges and provide insights into future research directions.

## Principle of electrochemical dealloying

Dealloyed porous materials provide several advantages over other fabrication techniques, particularly due to their tunable pore structure. Highly uniform porosity can be developed by dealloying from wide range of precursor including amorphous alloys, solid solutions and intermetallic compounds depending on the initial alloy microstructure. These consistent pore structures reduce pathway tortuosity, resulting in faster kinetics and better electrode material utilization [8]. Additionally, homogenous pores enable higher electrode material loading which significantly boosts areal capacitance and energy density. The mechanical stability imparted by uniform porosity ensures longevity that prevents electrode degradation during repeated charge-discharge cycles [9]. Voltage hysteresis during cycling also can be minimized by uniform pore sizes, which is crucial for effective energy recovery and decreased energy dissipation [10].

However, in comparison, achieving similar uniformity with other methods poses significant challenges due to the complexity of operations required. For the first example, additive manufacturing excels in fabricating intricate and customizable porous structures with high geometric complexity [11]. However, it struggles to consistently produce gradient porosity or nano- to micro-scale pore, primarily due to computational and printing control limitations [12]. Challenges such as excessive binder inclusion, resin optimization requirements [13] and inadequate process planning software [14] further constrain its ability to achieve uniform pore distribution. Second, template-based methods including sacrificial and replica techniques allow controlled pore size and distribution but are limited by the complexity of template preparation and processing conditions. These methods are not always direct that lead to variations in pore filling and potential restructure after template removal [15]. While they can achieve high porosity, they lack adaptability for specific structural designs. Replica methods are constrained by the inherited pore structures, offering limited design flexibility [16]. Third, the sol-gel process is another versatile technique, enabling the fabrication of macroporous and microporous materials with tunable structures. However, ensuring uniformity in pore size and distribution is challenging, as the process relies on external additives for structural stabilization [17]. The removal of these additives and subsequent sintering steps often introduce variability in the final structure. Maintaining stability during the gelation and drying phases can be problematic, leading to cracks and structural inconsistencies [18]. Similarly, the hydrothermal method is effective in synthesizing porous materials with hierarchical structures and incorporating functional groups [19]. However, it is highly sensitive to reaction conditions such as temperature, pressure and precursor concentration, making precise control essential [20]. Achieving consistent porosity requires meticulous management of nucleation and growth, which can complicate scalability. Overall, while alternative methods such as additive manufacturing, template-based approaches, sol-gel processes and hydrothermal techniques offer specific advantages, they are often hindered by complexities in achieving uniformity and precision. In contrast, dealloying uniquely combines structural tunability, simplicity and process efficiency, making it ideal choice for advanced applications in energy storage, catalysis and material science.

In recent decades, dealloying has been widely deemed as one of the most promising strategies to fabricate free-standing porous materials due to its straightforward approach that allows for control over pore sizes ranging from nanometer grade to micrometer grade [21]. Dealloying is described as corrosion process that involves selectively dissolving metal from the parent alloy and allow the remaining metals to reorganized, thus result in the formation of nanoporous surface. Nonequilibrium behaviour at the interface between alloy and etchant shows the fundamental of dealloying [22]. Different methods such as chemical, electrochemical, solid-state [23], vacuum sublimation [24], freeze-drying [25], metallize template sacrificial [26] and plasma etching reaction [27] have been introduced. Apart from that, selective oxidation or evaporation at higher temperatures and interactions with molten salts or liquid metals provide further versatility in creating advanced dealloyed porous materials [28]. It is important to understand various dealloying techniques in order to determine the best approach based on the required properties of material, performance, compatibility, cost, scalability and influence on the environment. Table 1 summarized the salient comparison between several dealloying methods. Review of the literature suggests that the most auspicious method for porosification is electrochemical dealloying. This method utilizes the difference in chemical reactivity that offers high reproducibility and does not require high temperature or pressure that commonly found in traditional wet-chemistry procedures for nanomaterials [29]. Scaling up this dealloying process for mass manufacturing is relatively simple to create nanoporous materials with any desired form such as bulk monolithic [30], thin films [23], melt-spun ribbons [31], compressed metallic powder [32], foams [33], sponges [34] and nanoparticles [35]. Understanding the corrosion and degradation behaviour is crucial for controlling and designing anode with desired properties of surface structure, especially for energy storage applications.

**Table 1.** Comparison between dealloying methods.

Dealloying methods	Chemical/electrochemical	Liquid metal	Solid-state dealloying	Vapor phase/vacuum sublimation
Working principle	Difference in chemical activity of the alloy components	Difference in enthalpy of mixing between each element of the precursor alloy with a molten third element	Difference in enthalpy of mixing between the elements of precursor alloy and dealloying medium	Difference in saturated vapor pressure of the alloy components
Dealloying medium	Aqueous electrolyte	Liquid metal	Solid metal	Atmosphere
Advantages	Mature technology Easy processing procedure Cost-effective method for large scale production Better controlled dealloying process	Compatible with active metals Produce well-defined porous structures High selectivity for the targeted dealloying component	Compatible with active metals Produce finer ligament size Minimize ligament coarsening effect during dealloying	No chemical etching Free of liquid waste Evaporated elements can be recycled Produce highly uniform porosity
Disadvantages	Requires precise control of dealloying conditions Produce chemical wastes Required corrosive chemical Inapplicable to active metals	Time-consuming Need additional etching process to expose the porous structure High cost due to temperature constraints	Applicable only to thin-film geometry or interface-driven process Dealloying process is slower than liquid-based methods	High cost due to complex setup for high-temperature and high-vacuum conditions Not suitable for close-saturated vapor pressure materials

Electrochemical dealloying can be initiated either by applying external electric current or by spontaneous chemical reaction between the etchant and the parent alloy under condition of free corrosion [1]. Although sometimes referred to as chemical dealloying, it is essential to note that the underlying mechanism is fundamentally electrochemical. Electrochemical and chemical dealloying methods yield materials with distinct pore structures, shaped by their respective mechanisms. During electrochemical dealloying, structural evolution involves complex processes such as bulk and surface diffusion, ion transport and electrochemical reactions, which can lead to the formation of nested porous networks. By varying the applied potential, pore size and distribution can be finely tuned. This method may also induce crystallization in certain materials that influence the formation of ligaments and grain regions, which in turn affects the electrochemical properties of material [36]. In some cases, electrochemical dealloying shows suppressed reordering processes behind the etch front which can be attributed to the formation of oxides [37]. Additionally, the lower surface diffusivity during electrochemical dealloying compared to chemical dealloying affects the coarsening mechanism and the final pore size [38]. In contrast, chemical dealloying produces uniform porosity through gradual and even dissolution, leading to bicontinuous networks. The pore size increases with extended dealloying time and can be controlled by adjusting the concentration and duration of chemical solution. Chemical dealloying can also result in more complex structural formations such as dendritic structures which are not typically observed in other dealloying methods [39]. However, this process may leave behind residual elements that impact the final porosity and composition of material [40].

Among varieties of etchant candidates, acidic or alkaline solution such as concentrated KOH, NaOH, HNO_3_, HF, HClO_4_, HCl and H_2_SO_4_ are commonly used to selectively remove the active metal component from the alloy. However, these traditional etchants pose significant environmental and health risks due to their toxicity and corrosive nature [41]. Careful management is required because the use of these etchants produces chemical waste and pollution. Future development of etchant treatment in etching machines should focus on integrating advanced regeneration technologies to enhance the sustainability of producing porous materials. Emerging technologies such as electrolytic process and membrane-based system offer promising solutions by enabling the regeneration of etching solution and recovery of valuable metal [42]. For instance, electrolytic approaches combine anode oxidation of sulphates with cathodic reduction of metals like copper, achieving closed-loop and zero emission operations while significantly lowering chemical consumption and operational costs [43]. Furthermore, advanced membrane technologies further enhance economic viability in etching processes. Nanofiltration membranes have demonstrated high rejection rates of copper ions from sulfuric acid waste solutions which enable long-term recycling of etching solutions [44]. Likewise, diffusion dialysis using anion exchange membranes efficiently recovers acids from industrial effluents in energy-efficient and environmentally friendly manner [45]. Together, these technologies contribute to minimize waste and foster circular material utilization. To deepen the understanding of etching mechanisms, combining simulation models with flow cell experiments provides valuable insights. Optimizing the supply of hydrogen ions to the precursor surface, particularly by balancing weak and strong acid dynamics, enhances etching efficiency and process precision [46]. Such approaches pave the way for improved control over dealloying kinetics and product quality.

Nitric acid (HNO₃) is currently one of the most widely utilized etchants in commercial dealloying processes. It is frequently employed for dealloying Ag-Au thin films, facilitating the formation of porous structures with precisely controlled volume fractions which are essential for development of porous metal catalysts [47]. In the same way, greener etchant of citric acid has proven effective for dealloying Mg-based metallic glasses to produce nanoporous Cu, Ag and Ni with sponge-like morphologies [34]. The capping effect reduces metal surface diffusion and promotes the formation of highly porous structures. However, while organic etchants including citric acid, lactic acid, oxalic acid, tartaric acid and succinic acid are emerging as environmentally friendly alternatives, their adoption remains limited by slower dealloying kinetics [48]. The oxidation of intermediate products can compromise both purity and efficiency. Optimizing post-treatment processes is therefore essential to achieve high-purity products and industrial scalability. From the cost perspective, regenerating acids offers significant economic benefits by reducing acid consumption and recovering valuable materials [49]. For example, hydrochloric acid regeneration can cut acid consumption by up to 50%, directly lowering operational costs [50]. Techniques such as replenishing etch baths with concentrated acids help maintain process accuracy and quality while reducing costs. The utilization of spent etching solutions for neutralization processes presents effective cost-saving measure by minimizing reagent expenses.

**Figure 1.**
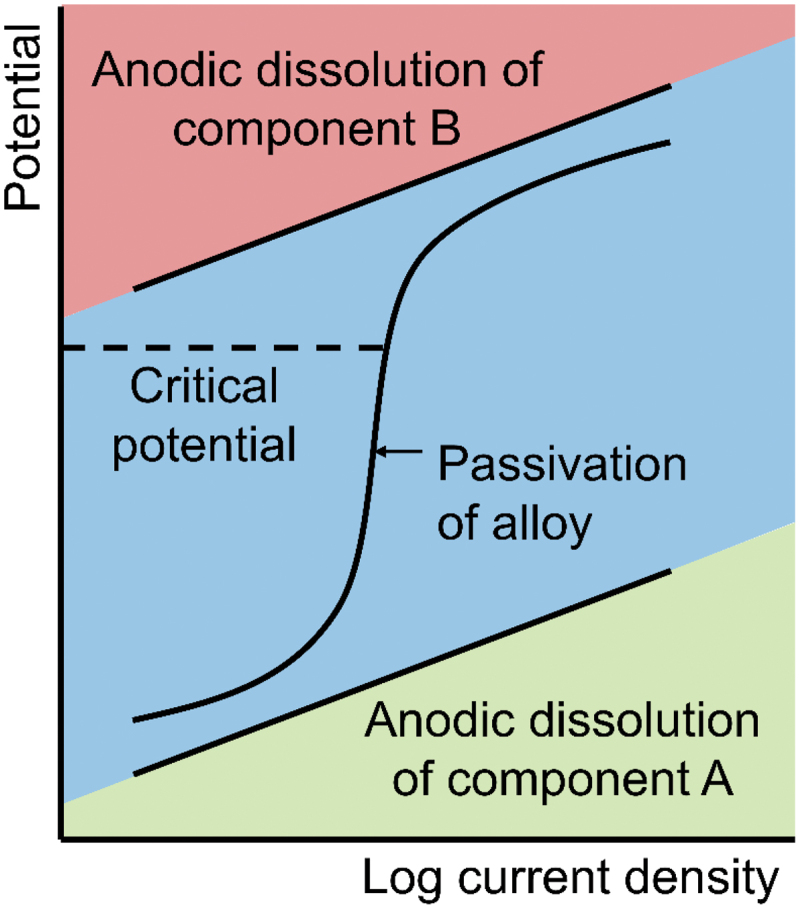
Schematic illustration of the anodic polarization curve for binary A-B alloy system, where a represents the less noble component and B represents the more noble component.

**Figure 2.**
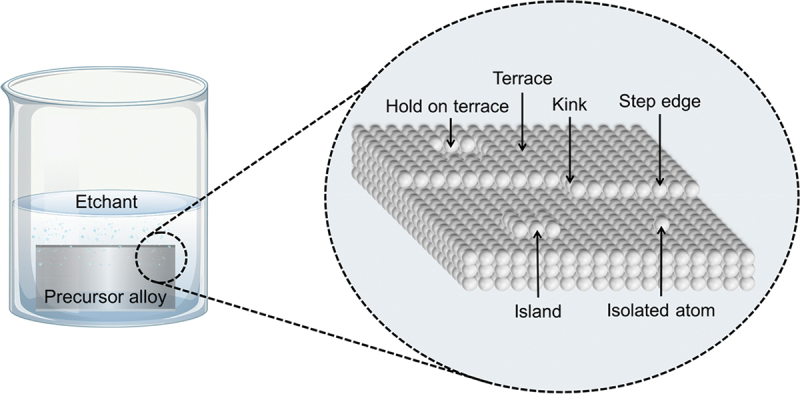
Atomistic hard-sphere model of the dealloyed porous structure.

The removal of active component is initiated when the electrochemical potential exceeds a composition-dependent critical potential [51]. This potential increases as the content of noble metal in the precursor master alloy increases [28]. Moreover, alloys with different crystal structures require distinct applied potentials for effective corrosion due to variations in their electrochemical properties [52]. However, when the potential falls below critical, alloy tends to remain planar and become passivated with more novel atoms. Based on Figure 1, the schematic anodic polarization curve of a binary alloy A-B illustrates the passivity range. During dealloying process, the remaining atoms rearrange themselves to occupy the spaces vacated by the dissolved atoms and lead to the formation of a three-dimensional bicontinuous nanoporous skeleton [29]. Dynamic interaction between the chemical etching of reactive species and the atomic rearrangement of inert species leads to the formation of final porous structure [53]. Initially, according to the atomistic hard sphere model as shown in Figure 2, less noble atoms that located at low coordination sites such as step-kinks and step-edge dissolve easily, while the remaining more noble atoms form a protective layer on the surface.

**Figure 3.**
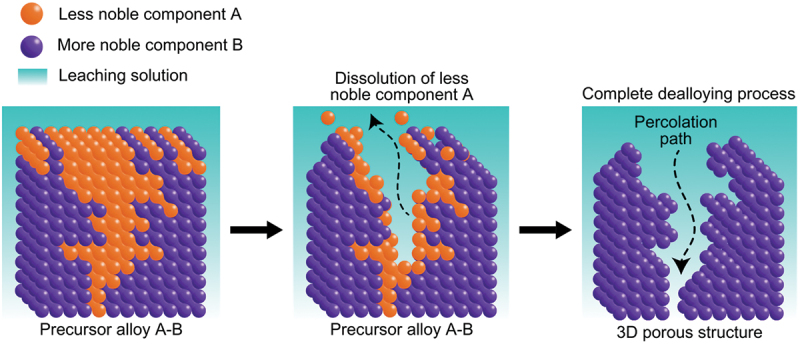
Schematic of electrolyte percolation pathways resulting from porosity development.

**Figure 4.**
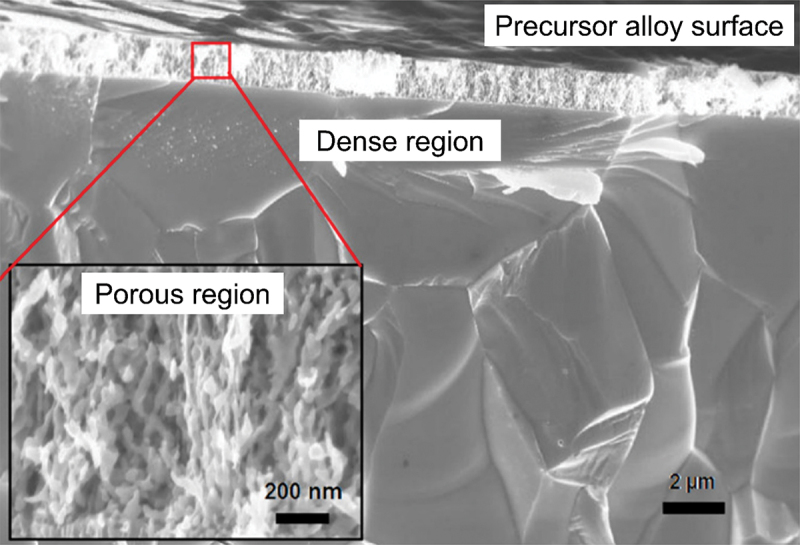
SEM image of boundary between the porous as-dealloyed region and the dense un-dealloyed region. Reproduced with permission from ref [48]. copyright © 2012 Elsevier.

However, the slowest step in this process is the gradual dissolution of atoms from high coordination sites such as terraces which occurs layer by layer [54]. Step edges around the dissolving area recede as vacancy clusters expand. As the step edges recede, more noble species tend to diffuse along with them instead of remaining as isolated atoms. Imperfections like step edge cause electronic perturbations on the smoother area of terrace and lead to variations in the surface electronic behaviour [55]. The dissolution of atom out of a terrace position will become thermodynamically possible at higher overpotentials. However, the diffusion velocity may be slower than the rate of step etching [29]. This instability causes the steps to break up into islands passivated by the noble metal at their perimeters. As more layers are dissolved, more noble metal atoms are needed to passivate the step edges of these islands [56]. Over time, the islands can be undercut because there will be insufficient noble metal atoms to sufficiently passivate them as their perimeters grow and the distance between them decreases [57]. This allows the porosity to propagate into the bulk alloy and increases the nominal surface area. As shown in Figure 3, the pores provide pathways for the percolation of electrolyte to extend the porosity. Dealloying velocity is determined by the rate of corrosion which can be observed through the thickness of the resulting porous structure. During dealloying process, the reaction front progresses uniformly parallel to the alloy surface and establishes a clear boundary between the porous and dense regions as illustrated in Figure 4. Wang et al. observed that within the remaining dense zone, the crystal grains and boundaries are well-defined [48]. This observation suggests that dealloying occurs at a consistent rate in perpendicular direction to the surface.

The bicontinuous porous ligament-void structure is formed by simultaneous action of surface diffusion of the more noble elements and dissolution of the active components from the uppermost layer. The relationship between dissolution rate and surface diffusivity governs the overall kinetic and behaviour of dealloying process [58]. To advance this field, it is imperative to further investigate the fundamental mechanisms of dealloying with particular focus on the roles of bulk diffusion and interfacial processes. Real-time in situ observations of pore evolution during dealloying can offer invaluable insights into these dynamic processes, enabling deeper understanding of material transformation at the atomic level. By integrating cutting-edge imaging technologies, fractal analysis and advanced simulations, researchers have the opportunity to uncover novel dealloyed porous material.

## Effects of dealloying conditions

In many cases, the resulting porous structure in electrochemical dealloying can be anticipated based on the driving force exerted by the applied potential to induce chemical reaction. However, the successful preparation of well-defined nanoporous structures remains definitively challenging due to complex interplay between alloy designs and dealloying kinetics. The dynamic nature of alloy dissolution and the subsequent reorganization of remaining atoms introduce significant variability, making it difficult to achieve precise and reproducible pore architectures. To optimize porosity in the final structure, precise control over various dealloying is critical. Achieving this level of precision is crucial to fine tune the pore size, distribution and overall morphology to meet specific application requirements. Table 2 provides comprehensive overview of recent studies that have successfully manipulated dealloying conditions to control the pore structure, highlighting key findings and experimental techniques that have led to advances in nanoporous material design. In order to choose suitable alloy precursors, careful consideration of the elemental composition is essential. This typically involves the combination of at least one less noble element and one more noble element. The less noble element must undergo selective dissolution under appropriate chemical or electrochemical conditions, while the nobility gap between the two elements ensures the formation of stable porous structure [69]. Furthermore, the corrosion potential and reactivity of the less noble element must align with the properties of etching solution or applied potential range to enable controlled dealloying [70]. Economic and environmental factors also play critical role in the selection process, favouring precursors composed of abundant, cost-effective and non-toxic elements to support scalability and sustainability. Aluminium is commonly used alloying element due to its low cost, ease of alloying and good amphoteric nature [48]. The ability to react and dissolve readily in both acidic and alkaline etchants makes it ideal candidate for selective removal during the dealloying process [71].

**Table 2.** Effects of various dealloying conditions on the final porous structure.

Type of precursor	Alloy component	Etchant	Dealloying parameter	Findings	Ref.
Multi-component alloy	Zr_50_Cu_21_Ni_13_Al_9_Ti_7_ (at.%)	Choline chloride-thiourea (ChCl-Thi)	Electrochemical dealloying at 75°C with potentials of 1 V, 2 V, 3 V and 4 V (vs. SHE) for 30 min, 45 min, 1 h and 2 h	The optimal dealloying parameters were 3 V and 2 h duration time which produced well-developed porous structure ranging from 20 to 180 nm	[59]
	Zr_51_Cu_21_Ni_14_Ti_8_Al_6_ (at.%)	0.1 M HCl + 0.02, 0.05, 0.1, 0.15 and 0.2 M NaF	Electrochemical dealloying at 10°C with potentials of 0.4 V (vs. SHE) for 220 s	Increasing NaF concentration to 0.15 M produces uniform 3D porous structure with pore diameters of 0.2–0.6 μm, while at 0.2 M NaF, the structure remains 3D but with larger and more uneven pores	[60]
	Cu_50_Zr_40_Ni_5_Be_5_ (at.%)	0.05 M HF +0.5 M H_2_SO_4_	Chemical dealloying at 25°C, 50°C and 75°C for 4 h, 8 h and 12 h	Increasing dealloying time and temperature leads to larger and more uniform pores, however, excessively high temperatures can cause pore coarsening and thicker ligaments	[61]
Ternary alloy	Cu_45_Al_45_Ti_10_ (at.%)	0.2 M, 0.4 M, 0.6 M and 0.8 M HCl	Chemical dealloying at 25°C, 40°C, 55°C and 70°C for 60 min, 120 min, 180 min and 300 min	The average ligament width increased with higher HCl concentration, higher dealloying temperature and longer dealloying time	[62]
	Mg-Cu-Sn	5 wt.% HCl and 1 wt.% TA	Chemical dealloying at 25°C until no bubbles emerged	5 wt.% HCl caused more trans-granular cracks and produced ligament-channel structure with 20–40 nm ligament size due to faster Mg dissolution compared to 1 wt.% TA	[63]
	Al_70_Si_18_Ge_12_, Al_75_Si_15_Ge_10_, Al_80_Si_12_Ge_8_, Al_85_Si_9_Ge_6_ and Al_90_Si_6_Ge_4_ (at.%)	5 wt.% HCl	Chemical dealloying at 60°C with constant magnetic stirring for 24 h	As the Al content increases from 70% to 75%, coral-like architectures form that increase the surface area, however, increasing Al content to 85% reduces the porosity due to fragmenting	[64]
Binary alloy	Cu-Zn	0.1 mol/L HCl	Chemical dealloying at 30°C, 60°C, 70°C, 80°C and 90°C for 5 h	As the dealloying temperature increases, the diffusion rate increases which strongly affects copper atomic diffusivity and correlates with increased ligament coarsening	[65]
	Cu-Ga	0.1, 0.2 and 0.3 M HNO_3_	Chemical dealloying at 25°C, 50°C, 70°C and 90°C for 4 h	Higher dealloying temperature increase diffusivity, leading to greater ligament coarsening, while higher leaching solution concentration improves pore connectivity	[66]
	Cu-Zn	5 M and 7 M HCl	Chemical dealloying at 20°C, 35°C and 50°C for 280 h, 120 h, 35 h and 100 h	Higher dealloying temperatures and HCl concentrations increase ligament and pore sizes due to enhanced Cu diffusion rates	[67]
	Al_50_Sn_50_, Al_65_Sn_35_ and Al_80_Sn_20_ (at.%)	5 wt.% HCl	Chemical dealloying at 70°C for 120 min	The average pore size increases with higher Al content in the precursor alloy, indicating that the porous structure is formed by directly removing the Al phase from each Al – Sn alloy	[68]

**Figure 5.**
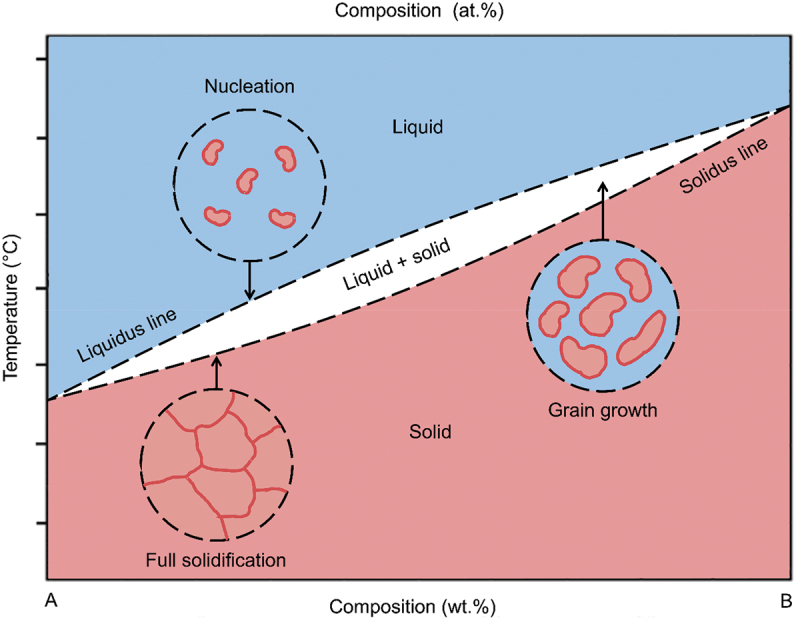
Schematic representation of phase transitions in the binary isomorphous alloy system.

The quality of porosity can be controlled by selecting suitable alloy compositions in binary, ternary or multicomponent systems with differences in crystal structure, grain size and orientation [72,73]. In general, the ligaments size and pores decrease proportionally with the increase in noble component in the precursor alloy [74]. However, the precursor alloy has different crystallographic epitaxial relationships and its solid solution solubility varies depending on the constituents. This characteristic influence the evolution of pores which ultimately shapes the final properties of porosity and ligament structure. Most attention has been devoted to the dealloying of Ag – Au system [48]. This alloy exhibits complete solubility across the entire compositional range due to complete miscibility of the host element and alloying element in both molten metal and crystal phases [75]. The isomorphous binary system has smooth transition from liquid to solid solution without the formation of any new phases as shown in Figure 5. This complete solubility enables the formation of homogeneous alloys that results in a single solid-phase microstructure.

**Figure 6.**
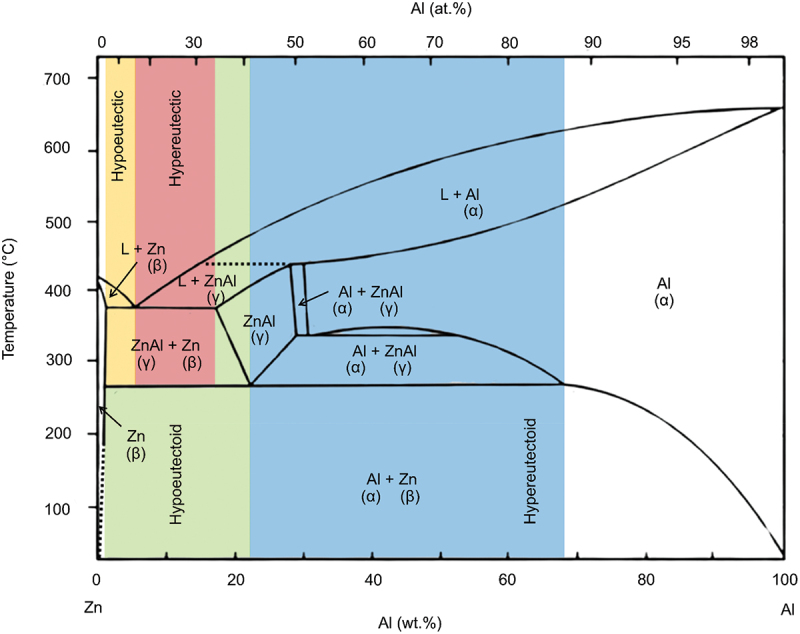
Phase diagram of the zinc-aluminium (Zn-Al) alloy system.

Conversely, alloy system such as Cu-Zn, Zn-Al, Cu-Al, Al-Si, Al-Au, Al-Pd, Al-Pt, Mg-Cu and Al-Ni differ significantly because they fall under the category of partially soluble limit alloy. Therefore, it exhibits differences in structure and dealloying behaviour due to the formation of various ordered phases depending on the composition. Different phases in alloy exhibit dissimilar electrochemical properties due to different crystal structures. This allows for the creation of porous structure across multiple length scales by utilizing multiphase parent alloys. In systems with limited solubility, precursor alloys usually consist of one intermetallic phase and one solid-solution phase, as seen in many binary eutectic systems. Dealloying can occur in either phase to promote the formation of diverse porous architectures. For example, Zn-Al system shows two terminal phases, (Al) and (Zn) phases, which are Al- and Zn- rich solid solution phases respectively as shown in Figure 6. Zn-Al alloy consist of Zn and Al matrixes without intermetallic compound [76]. The Al-rich phase, (Al) is expected to be leached out during dealloying to produce nanoporous Zn surface in the alloy. Theoretically, 78 wt% Zn alloy can produce 100% eutectoid structure of (Al) and (Zn) phases in the lamella structure. Meanwhile, 95 wt% Zn alloy is expected to produce eutectic structure of (Al) and (Zn) phases in the lamella structure. The fine and uniform distribution of eutectic structure ensures that when aluminum is selectively leached out, the resulting pores are evenly distributed throughout the zinc matrix. Indeed, the regularity of the alternating lamellar structure leads to consistent pore distribution after dealloying [77]. This will result in more pronounced and interconnected porous morphology.

Another type of parent alloy is the multicomponent single-phase alloy. The constituents have different electrochemical potentials that lead to different dissolution rates during dealloying. High-entropy alloys (HEAs) have garnered attention among the researcher due to their enhanced mechanical properties and unique microstructures. The most significant limitations of traditional nanoporous metals dealloyed from normal precursor alloys is their inherent brittleness, which restricts the practical applications [78]. In contrast, HEAs provide superior strength, ductility and fracture toughness which offer a potential solution which make them attractive candidates for the development of more robust porous metallic materials [79]. Recently, combination of the long-range disordered atomic structure of amorphous alloys with the compositional complexity of high-entropy alloys has led to the development of high-entropy amorphous alloys (HEAAs) [80]. These materials possess very distinctive physical, chemical and mechanical properties, offering significant potential for dealloying applications. Physically, HEAs exhibit extraordinary strength and hardness, driven by significant lattice distortion and the synergistic cocktail effect where the interaction of multiple elements enhances overall performance [81]. Their remarkable thermal stability allows them to maintain structural integrity at elevated temperatures, making them ideal candidates for extreme environments such as high-temperature energy storage systems [82]. Additionally, HEAs demonstrate exceptional corrosion resistance, attributed to their complex atomic configurations which stabilize solid solution phases and limit the formation of galvanic couples in aggressive environments [83]. Chemically, high mixing entropy inherent to HEAs stabilizes single-phase solid solutions and suppresses the formation of brittle intermetallic compounds, ensuring uniform structure and improved reliability [84]. Their highly tunable surface chemistry further enables the development of efficient catalytic materials. By tailoring elemental compositions, HEAs can optimize adsorption sites and electronic properties, making them promising candidates for applications in catalysis, energy conversion and chemical processes. Mechanically, HEAs show balance of strength, ductility and toughness, overcoming the long standing trade-off between strength and brittleness often seen in conventional precursor alloys [85]. The multi-element design provides diverse deformation pathways and delayed dislocation movement, ensuring excellent resistance to fracture even under high stress. Furthermore, HEAs offer superior radiation resistance due to their complex atomic structures, which suppress defect migration and accumulation, making them particularly suitable for nuclear power energy storage applications [86]. Their high wear resistance enhances the durability in applications requiring prolonged operation under abrasive conditions. The unique characteristics of HEAs create immense potential for dealloying applications. The inherent compositional metastability of HEAs that arises from their high configurational entropy promotes controlled phase separation and the formation of intricate nanostructures during dealloying [87]. This process can generate highly porous architectures with precisely tailored pore sizes, surface areas and morphologies, which are invaluable for catalysis and energy storage. Although their exceptional corrosion resistance may initially limit dealloying kinetics, this challenge can be mitigated through careful tuning of etching parameters, enabling the fabrication of nanoporous HEAs with tailored functional properties. In summary, unparalleled combination of strength, stability and tunability makes HEAs the transformative material class, not only for structural applications but also for cutting-edge dealloying techniques. Their ability to form complex nanostructures through controlled leaching places them at the forefront of innovations in advanced energy systems. By leveraging the unique physical and chemical complexity of HEAs, researchers can unlock new opportunities for designing high performance materials tailored for next generation applications.

**Figure 7.**
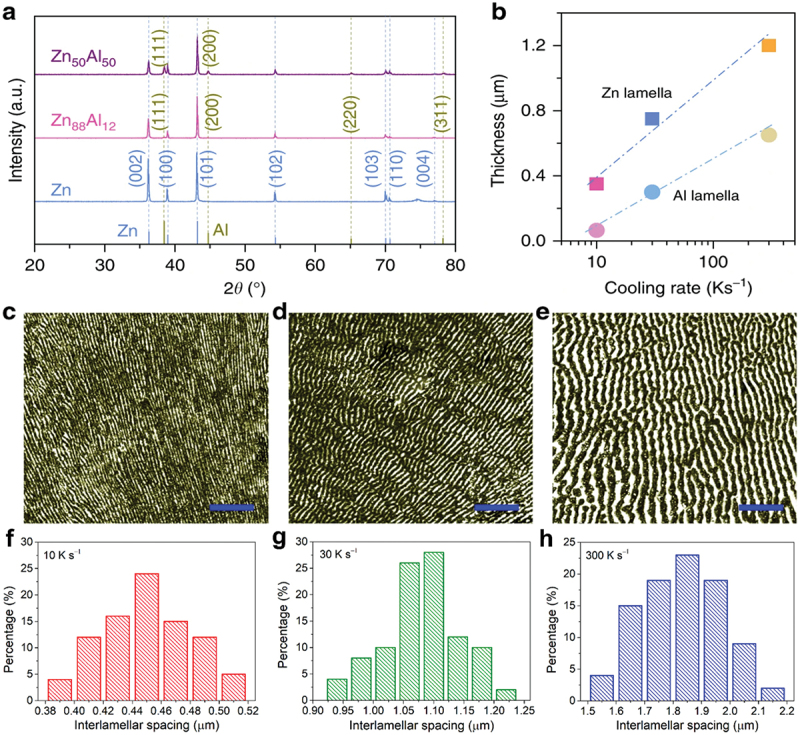
a) XRD patterns of Zn-Al alloys, b) correlation between the lamella thickness in eutectic Zn_88_Al_12_ (at.%) alloys and cooling rates, optical micrographs of eutectic Zn_88_Al_12_ alloys with lamellar spacing of c) ∼450 nm, d) ∼1050 nm and e) ∼1850 nm (scale bar: 10 μm), interlamellar spacing distribution for eutectic Zn_88_Al_12_ alloy at cooling rates of f) 10 Ks^− 1^, g) 30 Ks^− 1^ and h) 300 Ks^− 1^. Reproduced with permission from ref [88]. copyright © 2020 Springer nature.

In addition, the growth of porous structures is highly dependent on the distribution and size of each phase in the parent alloy. Modifying the microstructure of a solidified alloy can be achieved through appropriate control of heat treatment or solidification process. For example, by adjusting the cooling rate of eutectic Zn_88_Al_12_ (at%) alloy, the thickness of lamellae and interlamellar spacing were successfully determined as shown in Figure 7(b) [88]. At high cooling rate of ∼300 Ks^−1^, the eutectic lamellae coarsen into more defined structures with lamella spacing of ∼1850 nm (Figure 7(e)). However, in contrast with Li et al. the fine lamellae in hypereutectic Al_75_Cu_25_ alloy coarsen into larger eutectic structures when subjected to slower cooling rate of 2 °Cmin^− 1^ [89]. Despite these differences in solidification behaviour, the resulting porous structure generally mirrors the phase structure present in the precursor alloy. This relationship highlights the importance of phase formation during solidification, as it plays critical role in deciding the final porous structure. To further refine pore development, additional technique such as unidirectional solidification can be employed to enhance phase distribution control. This method offers the ability to create well-aligned pore channels by orienting the growth of the phases parallel to the heat gradient [90]. This innovative approach opens new pathways to modify porous architectures, making them suitable for a broad range of advanced applications.

When precursor master alloy is exposed to aggressive electrolytes for extended periods, selective dissolution is not achieved [91]. This happen because the high reactivity of electrolyte leads to simultaneous dissolution of both matrix and alloying elements. Prolonged exposure can also lead to the formation of passivation layers, which act as barriers to further dissolution and prevent the targeted removal of less noble elements. A key study by Zhu et al. found that the BET, real and electroactive surface areas are reduced by 60%, 50% and 30% respectively, when the concentration of HCl increased from 5 to 7 M at the same dealloying temperature of 20°C [67]. This is because a stable particle is formed at the solid-liquid interface when more noble atoms migrate due to increase in the atomic diffusion rate. Achieving appropriate concentration to balance surface diffusion and dissolution is necessary to develop the desired nanoporous design.

**Figure 8.**
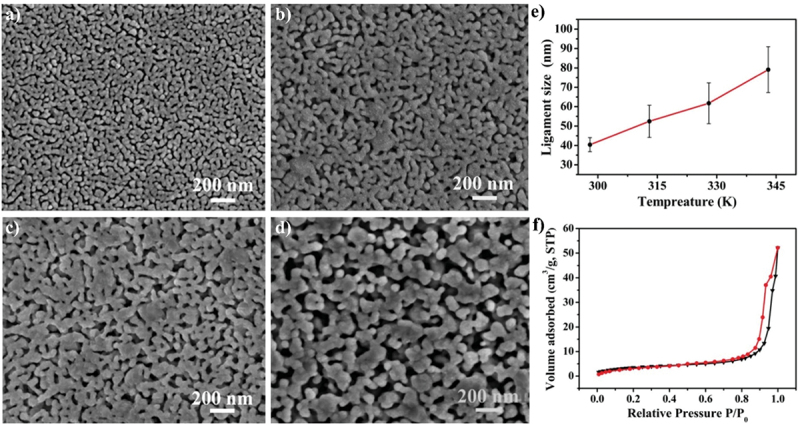
SEM images of nanoporous Cu produced by dealloying Cu_45_Al_45_Ti_10_ amorphous ribbons in 0.4 M HCl at temperatures of a) 298 K, b) 313 K, c) 328 K and d) 343 K, e) the relationship between temperature and ligament size and f) nitrogen adsorption-desorption isotherm. Reproduced with permission from ref [62]. copyright © 2020 Taylor & Francis.

Furthermore, kinetic factors such as temperature can influence porosity evolution. High dealloying temperature cause ligament coarsening and increase the fracture elongation [65]. This coarsening effect compromises the uniformity and fine structure of pores which are essential for design requirement of anode [66]. Porosity and ligament width increase as the dealloying temperature increase. Figure 8 shows the morphology of nanoporous Cu after dealloying at different temperature. From SEM images, the distribution of pore and ligament size can be quantitatively determined by using ImageJ. Qian et al. observed that Cu ligament size increased from 40 nm to 79 nm as dealloying temperature raised from 25 to 70°C (Figure 8(e)) [62]. The high specific surface area of 12.69 m^2^/g was attributed to the unique size-dependent properties of ligaments within the porous structure (Figure 8f). Similarly, Scaglione et al. observed that Au ligament size increased from 77 ± 17 nm to 142 ± 43 nm as dealloying temperature increased from 20°C to 90°C [92]. Additionally, Tan et al. found that subsequent treatment including high temperature annealing can further coarsen the ligaments due to surface self-diffusion of the more noble atom [93]. As the result, the total BET surface area decreases from 6.4 m^2^/g to 1.8 m^2^/g, while the total pore volume contracts from 0.04 cm^3^/g to 0.008 cm^3^/g.

Furthermore, adjusting the applied potential and current during electrochemical dealloying enables precise control of the chemical etching rates. Zhang et al. conducted a study to produce higher purity and porosity metal by dealloying under pulsed voltage waveforms [53]. As the result, larger specific surface area and smaller ligament are produced compared to dealloying with constant voltage. Moreover, the sizes of pores and ligaments increased when the applied potential is higher [94]. Dealloying at lower potential leads to the formation of smaller pores due to the more controlled reaction kinetics and less aggressive dissolution process [95]. At lower potential, the driving force for dealloying reaction is reduced. This can lead to slower reaction rate and more controlled removal of the less noble component. The slower rate allows for finer control over the pore structure, thus result in smaller sized pores. On the other hand, excessively high potential leads to the formation of granular structures due to passivation effect [59]. The passivation layer acts as a barrier that prevents the continued etching of the less noble component. Therefore, precise control over dealloying potential is critical for fine-tuning the process and achieving desired material properties. Exploring the kinetics of dealloying through the comprehensive set of collective dealloying parameters will unlock new pathways for optimizing the process, offering the potential for improved material efficiency in real-world applications.

## Properties of dealloyed nanoporous materials

**Figure 9.**
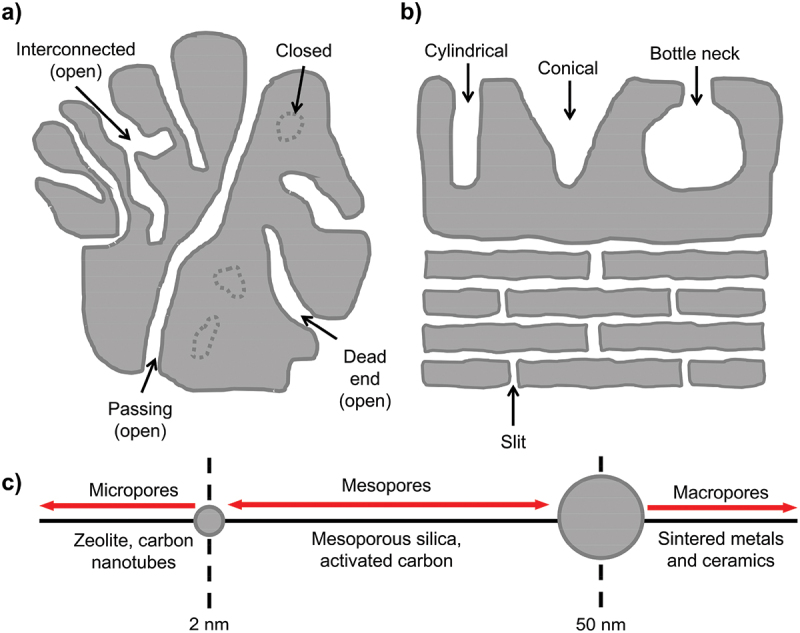
a) schematic illustration of porosity in open and closed pores, b) shapes of pores and c) classification based on their width according to the international union of pure and applied chemistry (IUPAC) standards.

Pores can be classified based on the size (micropores, mesopores and macropores) according to the International Union of Pure and Applied Chemistry (IUPAC) standards (Figure 9(c)). In materials science, the term ‘nanoporous’ is often used to describe pores that are on the nanoscale, although this is descriptive term rather than formal classification. Nanoporous materials can exist in zero-, one-, two- and three-dimensional (0D, 1D, 2D and 3D) in various morphologies including open (interconnected, dead-end or passing) or closed structures (Figure 9(a)). Additionally, the shape can vary such as cylindrical, conical or bottle-neck (Figure 9(b)). The evolution of pores can be present in either outer layer, inner layer or both. For electrode application, porous uppermost layer offers significant advantages due to high nominal surface area which enhances electrochemical performance. Meanwhile, the bulk solid core maintains excellent electrical conductivity, ensuring that the overall electrode retains good electrical performance [96]. Moreover, the core provides structural support to the pores that allows to withstand mechanical stresses and volume changes during cycling. Porous materials can be optimized by understanding the morphological, chemical, physical and mechanical properties.

The increased porosity enhances properties such as surface area, catalytic activity, conductivity and mass transport. High porosity can be achieved with longer dealloying times because it allows for more extensive removal of the less noble element [67]. However, prolonged dealloying can also reduce mechanical integrity due to surface oxidation, high volume shrinkage, stress-dealloying cracking and high internal stresses [97]. Admittedly, the formation of larger voids and thinner ligaments decreases structural connectivity and reduces the ability to withstand mechanical stress [98]. Changes in composition from dealloying can affect the strength, hardness, ductility, brittleness, toughness and rigidity. Micro scratch and nanoindentation test was conducted by Wang et al. to evaluate the hardness and strength of dealloyed surface [99]. As the result, the dealloyed nanoporous layer showed 82.0% and 90.1% reduction in elastic modulus and nanohardness, respectively, and required over 30% less scratching force. Additionally, during electrochemical cycling, structural degradation occurs on the anode due to repeated expansion and contraction which leads to mechanical fractures [100]. The crack propagation is driven by mechanisms such as coherency stress, residual stress and capillary forces between the alloy and the dealloyed structure [101]. Stress concentrations at thin ligament sites make these areas prone to cracking [102]. However, extended dealloying time at elevated temperatures can help release residual stresses, thereby reducing crack formation [103].

As the porous skeleton consists of interconnected networks that are joined by solid ligaments, the smaller ligaments induce stronger interfacial scattering which result in increased resistance [29]. Admittedly, porous metals have lower electrical conductivity compared to bulk counterparts [104]. The solid ligaments produce longer and more complex conduction paths that increase electron scattering. Charge flux travels along extended and tortuous pathway across the cross sectional area [105]. Additionally, the overall volume of metallic phase is reduced by the presence of dielectric pores which are less conducive to current flow. However, carrier density of nanoporous material remains unchanged despite variations in ligament size [106]. The smooth flow of charge carriers is facilitated by reducing microstructural defects like porosity [107]. This reduction minimizes disruptions to the flow of charge carriers, thereby decreasing electrical resistance. The resistivity can be evaluated by using the ordinary four-probe technique to understand the electric behaviour of dealloyed nanoporous material. In order to obtain high conductivity while maintaining high porosity, it is important to control the chemical composition and processing conditions [108]. Introducing specific dopants can improve the electrical properties of the base material. Apart from that, incorporating materials such as carbon nanotubes (CNTs), graphene, MXene or metal nanoparticles can enhance conductivity. However, pores modifications such as thermal treatment and the growth of CNTs involve complex synthesis processes that can be costly and impractical for large-scale manufacturing [1].

Nominal surface area of dealloyed nanoporous material is the critical property that needs to be systematically evaluated. Specific surface area can be measured by the Brunauer – Emmett – Teller (BET) method, while the Barrett – Joyner – Halenda (BJH) method is employed to measure pore size distribution, pore volume and pore diameter based on the adsorption branch of the isotherms [109]. Isotherm measures how the volume of gas adsorbed varies with gas pressure at a constant temperature. Hysteresis in gas sorption curves suggests the presence of mesopores and provides insights into the shapes of pores such as cylindrical, slits, conical and bottle neck [110]. It occurs when there is a difference between the adsorption and desorption branches of the isotherm. Adsorption occurs due to the forces interacting between solid surfaces and gas molecules. Chemisorption involves chemical bonding between the adsorbate and the surface that results in strong and irreversible interaction while physisorption involves weaker van der Waals forces. Nitrogen gas is the most commonly used adsorbate in gas adsorption studies. However, it is not completely inert and can have localized adsorption due to dipole interactions [110]. Argon gas is preferable for porosity measurements because it can access smaller pores more effectively than nitrogen. Despite that, the use of argon is limited by its availability and the higher cost. Consequently, nitrogen remains the standard choice for many applications. Apart from that, BET is limited to measure open pores, whereas small-angle neutron scattering (SANS) can account for both open and closed pore regions. In a study by Radlinski et al. SANS and BET data showed similar trend, but surface area values from nitrogen adsorption were consistently 2 to 6 times lower [111]. This difference is due to the ability of SANS to detect inaccessible pores.

SANS is non-destructive technique for characterizing closed pores. The method exploits differences in the neutron scattering length density between the material matrix and its surrounding environment [112]. To specifically detect closed pores, contrast matching is employed where the solvent or vapor with a scattering length density equivalent to that of the matrix is introduced [113]. As the solvent selectively fills open pores, it eliminates their scattering contrast, allowing the remaining signal to originate exclusively from the unfilled closed pores [114]. This approach provides clear and precise means of isolating and characterizing closed pore structures. The process begins with the exposure of sample to solvent vapor under controlled conditions. Through capillary condensation, the vapor gradually fills open pores as the pressure is systematically increased [115]. At each step, SANS measurements are performed to track the scattering signal. Closed pores remain inaccessible to the solvent and therefore retain their scattering contrast [116]. By analysing the scattering intensity and angular distribution, detailed information on the size, shape and spatial distribution of the closed pores can be derived. The incremental filling of open pores also enables differentiation between various pore sizes within the material. SANS offers significant advantages for closed pore detection. It is non-invasive technique that preserves the material structure, making it suitable for in-situ studies. Moreover, SANS is capable of probing wide range of pore sizes, from several nanometers to micrometers, providing comprehensive understanding of the pore network. The sensitivity to subtle changes in scattering contrast makes it particularly effective in distinguishing closed pores that are otherwise challenging to detect with conventional methods. To further enhance accuracy, SANS is often complemented by techniques such as nitrogen adsorption, mercury intrusion porosimetry [117] and helium pycnometry [112] to create more robust characterization framework. This is particularly critical for optimizing porous materials in energy storage system where the presence and behaviour of closed pores influence material performance. For example, closed pores enhance the low-voltage plateau capacity of hard carbon anodes in sodium ion batteries [118]. This is due to the adsorption of sodium ions at defect sites and the formation of sodium clusters within the closed pores. Future research should focus on developing advanced characterization techniques and predictive modelling to better understand the role of closed pores. Combining experimental methods like SANS, X-ray computed tomography and electron microscopy with computational simulations can provide deeper insights into the formation, distribution and influence of closed pores. Moreover, controlling the closed-to-open pore ratio through tailored manufacturing processes could enable the design of materials with optimized properties for energy storage applications. Addressing the current research gap on closed pores will unlock new opportunities for enhancing the functionality of porous metal materials.

**Figure 10.**
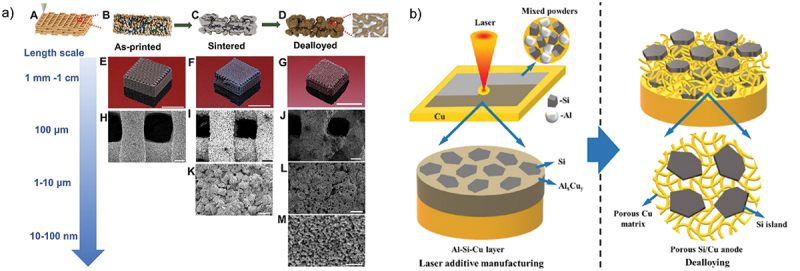
a) growth of the structural features at different length scales in the porous Cu by direct ink writing, thermal sintering and dealloying. b) illustration of the preparation of porous Si/Cu anode by combined laser additive manufacturing and dealloying. a) reproduced with permission from ref [126]. copyright © 2020 Elsevier. b) reproduced with permission from ref [129]. copyright © 2020 American Chemical Society.

Unimodal dealloyed porous design has limitation in electrolytic ion transport due to convoluted diffusion routes [119]. However, hierarchical architecture enhance mass transfer efficiency by integrating multiscale pores [120]. The integration of nano-sized and micron-sized pores significantly improve the transport properties [121]. Micropores play indispensable role in reducing material tortuosity and increase effective diffusivity while nanopores increase the specific surface area [122]. Creating hierarchical structures by combination of additive manufacturing and sequential dealloying has become an innovative technique. This process starts with 3D printing methods like powder bed fusion [123], selective laser melting [124], electron beam melting [125], direct ink writing [126] or material extrusion [127] to form upper level porous framework controlled by computer-aided design [128]. Then, electrochemical or chemical dealloying is employed to refine the structure into microscale or nanoscale porous features at the lower hierarchy level. This combined method offers enhanced control and efficiency in creating complex multiscale porous structures. As shown in Figure 10(a), Mooraj et al. presented three-step method to develop porous copper by direct ink writing, thermal sintering and dealloying in HCl, yielding trimodal hierarchy of macro-, micro- and nanoscale pores for diverse applications [126]. Besides, Cao et al. introduced porous Si/Cu anode using combined laser additive manufacturing and chemical dealloying, achieving high stability with 84.2% capacity retention after 100 cycles in full-cell Li-ion configuration (Figure 10(b)) [129]. However, porosification through additive manufacturing is seriously limited by low resolution and generally produces macro-sized pores. Scaling these techniques for commercial applications poses a significant barrier as current methods are confined to small-scale laboratory settings. In addition, matching the pore sizes in hierarchical porous electrode is complicated and emerging field. While qualitative descriptions are common, precise quantitative control over pore size distribution is still ambiguous.

Apart from that, geometric configuration of architected pores including the shape, orientation and alignment significantly influences the properties of porous materials [26]. In line with this, Miller et al. found that vertically aligned graphene oxide with cylindrical pores enhances wetting and ion accessibility during charge-discharge cycles [130]. This pore configuration also provides a stable scaffold for dealloyed nanoparticles to promote high catalytic activity in energy storage and conversion applications [131]. Thatikayala et al. reviewed on zeolite-based materials and noted that the current response during stripping analysis varies with different zeolite shapes [132]. Yu et al. reported that the unusual cage-like mesoporosity in graphitic-carbon frameworks contributes to superior electrochemical performance in Li-S batteries [133]. Additionally, radially oriented pore channels offer significant advantages for anode design. Jin et al. engineered hierarchically porous TiO₂ hollow microspheres with radially aligned nanorod chains that enhance electron flow during charge-discharge cycles [134]. Research on dealloyed porous materials has advanced particularly in the arrangement of pores including highly ordered or randomly arranged (stochastic). A periodic dealloyed pore structure with well-defined spatial arrangement and orientation enhances cyclic performance. For example, uniformly alternating dealloyed porous lamellar structure has been reported that promotes consistent pore distribution and results in excellent reversibility for the anode [77].

## Applications of dealloyed nanoporous anode materials

Electrochemical devices rely on critical functions such as surface area of electrode, mass transfer and electron conduction. Metallic porous materials excel in these roles due to their special 3D metal-and-void structures which include coherent ligaments for enhanced electron conduction and interconnected open pores for rapid mass transport. Their extensive surface area enables numerous charge transfer reactions, thereby improving the reversibility and cycle stability. Here, this section discusses the electrochemical applications of dealloyed nanoporous materials focusing on metal-ion batteries, supercapacitors, water splitting and fuel cells.

**Figure 11.**
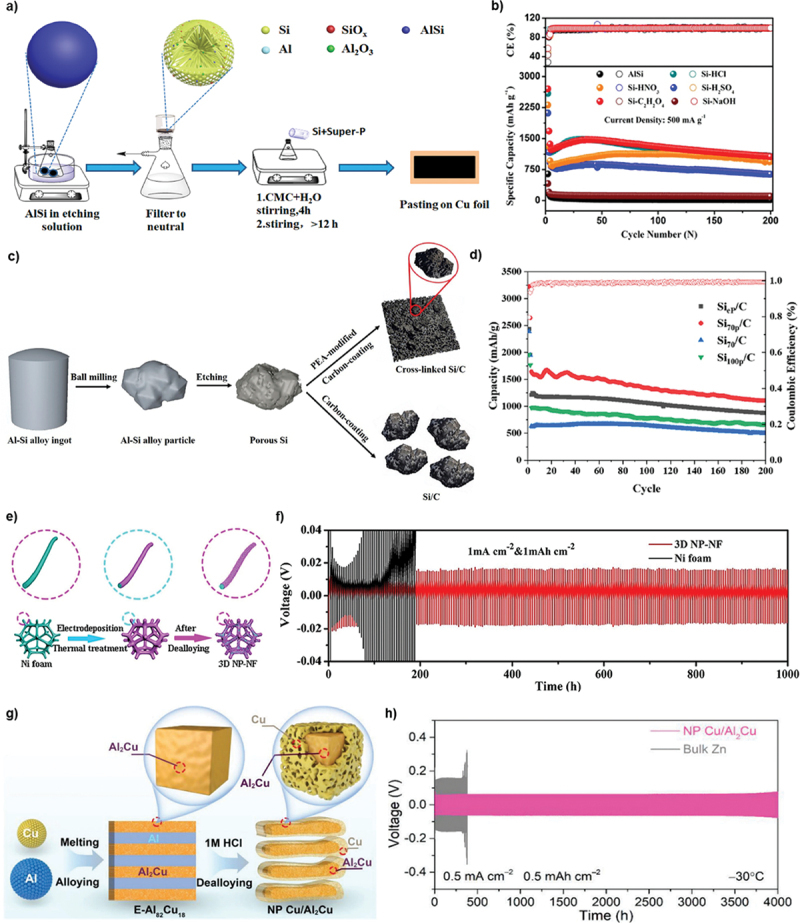
a) fabrication process of dealloyed porous Si anode and b) the specific capacity at 0.5 Ag^− 1^ for 200 cycles. c) preparation of dealloyed porous Si/C anode and d) the specific capacity at 0.2 C rate over 200 cycles. e) schematic of the synthesis process of 3D nanoporous Ni foam (NP-NF) and f) the voltage-time profiles at 1 mAcm^−2^ current density. g) evolution of lamellar nanoporous Cu/Al₂Cu anode and h) the voltage-time profiles at current density of 0.5 mAcm^−2^ in symmetric cell testing. a-b) reproduced with permission from ref [135]. copyright © 2019 Elsevier. c-d) reproduced with permission from ref [136]. copyright © 2019 Elsevier. e-f) reproduced with permission from ref [137]. copyright © 2024 Elsevier. g-h) reproduced with permission from ref [77]. copyright © 2024 John Wiley and sons.

Batteries work as electrochemical device that consist of anode and cathode which are separated by conductive electrolyte. The electrolyte can be in liquid or solid form to facilitate the transport of charge ions between anode and cathode. During discharge, the anode releases positive ions to the cathode and generate electric energy. Conversely, during charging, charge ions migrate from the cathode to the anode and prepare the battery for subsequent discharge. This cyclic process shows the functionality of batteries in storing and delivering electrical energy. Nanoporous materials play important role in battery systems, either as active electrode or as support structures that enhance conductivity. The high nominal surface area with interconnected pore networks makes them ideal for improving ion transport and charge storage capabilities. In LIBs, nanoporous materials increase energy density and rate performance by providing more active sites for electrochemical reactions. Admittedly, a significant study by Cao et al. demonstrated that porous Si anode chemically dealloyed in C₂H₂O₄ exhibited bicontinuous nanochannels with average diameter of 1–2 μm and achieved high specific capacity of 1063 mAh g^− 1^ after 200 cycles at 0.5 A g^− 1^ (Figure 11(a,b)) [135]. Similarly, Sun et al. reported that dealloyed porous Si/C anode maintained good reversible capacity of 1110 mAh g^− 1^ after 200 cycles at 0.2C rate (Figure 11(c,d)) [136]. Further contributions to this field include the work of Liu et al. who synthesized 3D nanoporous Ni foam (NP-NF) by chemical dealloying in 3 mol/L NaOH as illustrated in Figure 11(e). The removal of less noble Zn element resulted in large surface area of 1.71 m^2^/g and this anode demonstrated outstanding performance in symmetric cell tests, achieving extended lifespan of 1000 hours at 1 mA/cm^2^ current density (Figure 11(f)) [137].

Beyond that, ZIBs are widely explored due to the cost-effectiveness, non-toxicity, high stability and environmental friendliness compared to LIBs. However, they face challenges such as uncontrollable dendritic growth and issues related to zinc anode degradation which can impact the performance. Several anode modifications have been explored including alloying, surface coatings, the design of artificial solid electrolyte interface (SEI) layers and the incorporation of zincophilic materials. Despite these advancements, dealloyed nanoporous materials offer distinct advantages over planar metal foils. The unique porous structure can provide higher surface area with abundant nucleation sites for better electrodeposition reactions. For example, dealloyed nanoporous Zn_x_Cu_y_/Zn anode promotes uniform Zn deposition with 0 mV nucleation overpotential [138]. In addition, Meng et al. also produced lamellar nanoporous Cu/Al₂Cu anode through chemical dealloying in 1 M HCl as shown in Figure 11(g) [77]. The less noble Al component was selectively dissolved in HCl, leaving behind a robust Cu/Al₂Cu structure with well-defined lamellar features. This anode demonstrated excellent performance with long lifespan of 4000 hours at current density of 0.5 mA/cm^2^ in symmetric cell test (Figure 11(h)).

**Figure 12.**
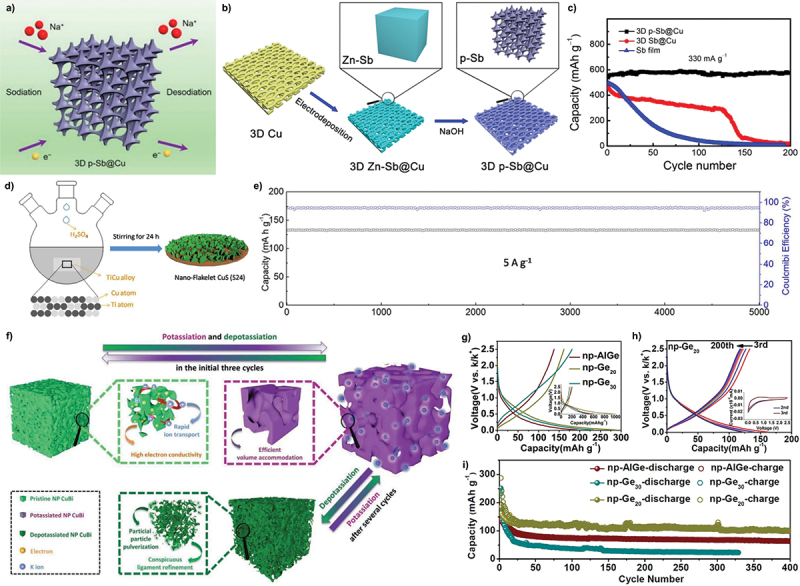
a) schematic of sodiation and desodiation processes at anode for SIBs. b) fabrication process of dealloyed 3D porous Sb and c) the comparison of cycling at rate of 330 mA/g for 200 cycles. d) preparation of chemically dealloyed porous CuS anode and the e) charge-discharge curves at 0.1 A/g. f) illustration of potassiation and depotassiation processes at anode during cycling in KIBs. g-i) electrochemical and cycling performance of dealloyed nanoporous Ge anode in the potassium-ion half cells. a-c) reproduced with permission from ref [141]. copyright © 2018 American Chemical Society. d-e) reproduced with permission from ref [142]. copyright © 2018 royal society of chemistry. f) reproduced with permission from ref [143]. copyright © 2020 John Wiley and sons. g-i) reproduced with permission from ref [144]. copyright © 2019 Elsevier.

Moreover, for the application on SIBs, dealloyed porous structures offer several advantages for enhancing the performance and durability. The large porous anode surface improves electrolyte infiltration and promote effective contact between the electrolyte and active material, resulting in enhanced charge and discharge capacity [139]. It also endows the anodes with high degree of capacitive contribution [140]. For example, dealloyed porous Sb anode demonstrated high reversible capacity of 624 mAh/g and maintained 575 mAh/g over 200 cycles (Figure 12(b,c)) [141]. Correspondingly, porous antimony/multi-walled carbon nanotube (De-Sb/MCNT) composite prepared via chemical dealloying exhibited high reversible specific capacity of 408.6 mAh/g and maintained 88% capacity retention after 330 cycles [145]. The interconnected porosity also facilitates efficient ion and electron transport which is crucial for high rate capabilities, as evidenced by porous Sb anode sustaining capacity of 132.6 mAh/g at high current density of 5 A/g after 5000 cycles (Figure 12(d–e)) [142]. Apart from that, dealloyed porous designs mitigate volume expansion during the sodiation and desodiation processes by incorporating optimized void spaces within their architecture (Figure 12(a)). Fang et al. justified the voids structures provide necessary flexibility to accommodate the volume variations caused by repeated sodium-ion insertion and extraction [140]. This feature helps maintain the structural integrity of anode material, reducing the risk of pulverization and enhances the cycling stability of SIBs. Admittedly, copper sulphide micro-flowers prepared by selective leaching exhibit high discharge capacity, attributed to their nanosheet composition which enhances sodium ion diffusion and accommodates volumetric changes [142]. By acting as mechanical stress buffer, the porous architecture minimizes material pulverization and enhances cycling stability [146]. For instance, porous Sb anode exhibited significantly reduced electrode swelling (63.96%) compared to its non-porous counterpart. Similarly, dealloyed nanoporous Bi-Sb alloy maintained exceptional structural integrity, achieving ultralong cycling performance over 10,000 cycles with negligible capacity decay [147].

In the same way, development of dealloyed porous anodes for KIBs focuses on addressing the significant volume changes that occur during potassiation and depotassiation processes (Figure 12(f)). Wu et al. engineered the 3D nanoporous CuBi anodes through chemical dealloying in NaOH solution and demonstrated the interconnected Cu and Bi ligaments help overcome slow potassium ion diffusion kinetics and electrode expansion during cycling, resulting in high discharge capacity and strong rate capability [143]. Large ionic radius of potassium often leads to sluggish reaction kinetics which can hinder the performance of anode materials, causing low reversible capacity and poor rate capability [148]. To address this, Cao et al. conducted dealloying method to grow dual-scale nanoporous architectures of np-InSb, which demonstrates superior reversible capacities and extended lifespan due to its unique dual-phase structure [149]. Similarly, selective leaching of aluminium has been applied to fabricate nanoporous germanium anodes, achieving stable capacities of approximately 120 mAh/g over 400 cycles compared to planar anode material for KIBs (Figure 12(g,i)) [144]. These advancements emphasize the value of dealloyed porous structures in overcoming the challenges and offer innovative pathways for high-performance energy storage solutions.

**Figure 13.**
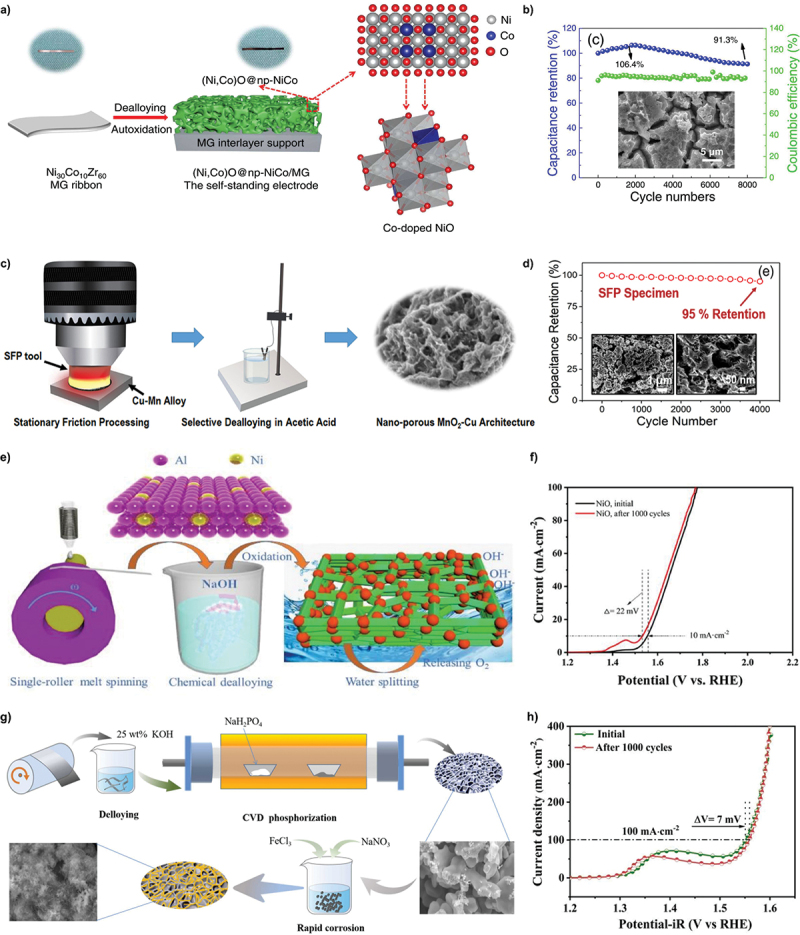
a) preparation of dealloyed nanoporous Ni-co, b) capacitance retention and coulombic efficiency after 8000 cycles. c) schematic of development of dealloyed nanoporous MnO_2_-Cu architecture and d) the cycle stability. e) fabrication process of chemically dealloyed nanoporous NiO and f) the linear sweep voltammetry curves before and after 1000 cycles. g) illustration of the synthesis process for nanoporous Ni_2.5_Co_2.5_-P substrate and h) the polarization curves before and after 1000 cycles. a-b) reproduced with permission from ref [154]. copyright © 2023 Elsevier. c-d) reproduced with permission from ref [155]. copyright © 2021 Elsevier. e-f) reproduced with permission from ref [156]. copyright © 2020 Elsevier. g-h) reproduced with permission from ref [157]. copyright © 2023 Elsevier.

For supercapacitors, nanoporous metals serve dual functions as capacitive materials and current collectors [150]. Supercapacitor has similar structure to the electrochemical batteries, consist of two electrodes, electrolyte and separator. Both electrodes are immersed in the electrolyte and separated by a separator layer. Pseudocapacitor involved fast reversible Faradaic reaction to store the energy electrochemically [151]. During charge and discharge process, electron transfer will occur between electrode and electrolyte interface by oxidation and reduction reaction. The high capacitance value can be achieved by increasing the specific surface area of anode [152]. Mirzaee et al. fabricated high surface area of nanoporous Ni – NiO electrode by electrochemical dealloying in 1 M H_2_SO_4_ and confirmed that 95.1% of the initial capacitance remain after 6000 cycles [153]. Likewise, one-step dealloying of Ni_30_Co_10_Zr_60_ (at.%) ribbons in 0.05 M HF solution leads to rapid etching of Zr, forming crack-like regions within the nanoporous structure (Figure 13(a,b)) [154]. Cycle testing reveals that this morphology endows the anode with capacitance retention of 91.3% after 8000 cycles. In a related study, Kumar et al. demonstrated nanoporous MnO₂-Cu structure achieved through selective dealloying of Mn which increased the contribution of diffusion-controlled processes and maintained capacitance retention of 95% over 400 cycles (Figures 13(c,d)) [155].

Electricity-driven water splitting has been adopted as viable method for producing clean hydrogen fuel from excess electrical energy generated by renewable sources. Hydrogen is highly regarded candidate for the green economy due to its high energy density and environmentally friendly production process. Therefore, scientists are extensively investigating water splitting as a primary method for hydrogen procurement. However, the efficiency of water splitting is hindered by OER at the anode which faces challenges from low stability, high overpotential and slow reaction kinetics. This bottleneck is largely due to the complex reaction intermediates involved in OER which limit the effectiveness of electrochemical water splitting. In order to boost reaction speed and reduce overpotential, catalysts with high intrinsic activity and optimized geometric structures are lauded. Dealloyed nanoporous materials have shown potential in this context as their large specific surface areas promote greater catalytic activity in OER. The design of porous surfaces plays a key role by exposing high density of catalytically active sites and leveraging interfacial effects. For example, Ren et al. dealloyed Ni_10_Al_90_ (at.%) precursor to form nanoporous NiO (Figure 13(e)) [156]. Accelerated degradation tests showed that after 1000 cycles of voltammetry, the overpotential decreased by 22 mV which demonstrated remarkable stability (Figure 13(f)). Similarly, Su et al. designed 3D porous Ni_2.5_Co_2.5_ alloy powder by dealloying in 25 wt% KOH, resulting in minimal potential shift of only 7 mV after 1000 cyclic voltammetry cycles (Figure 13(g,h)) [157]. Additionally, Li et al. fabricated continuous pore channels and Cu ligaments by dealloying Cu_28_Mg_72_ ribbon in 0.68 M HCl, achieving superior catalytic activity with excellent durability and maintained performance over 1000 cycles with minimal activity loss [158]. These studies underscore the importance of dealloying in creating nanoporous catalysts with enhanced stability and catalytic efficiency.

In photocatalytic energy storage systems, anode materials play important role in facilitating photoelectrocatalytic oxidation reactions by absorbing light to generate electron-hole pairs [159]. Dealloyed porous structure endows many advantages that enhances photocatalytic activity due to increase the number of reactive sites on large specific surface areas [160]. The interconnected pore channels facilitate efficient charge separation and transport of photogenerated electrons and holes, minimizing recombination and improve overall photocatalytic efficiency [161]. For instance, nanoporous CuS prepared by chemical dealloying reduces recombination rates where photogenerated electrons are efficiently transferred to trapping sites, improving energy conversion efficiency [162]. The porous design also improves light scattering and absorption, ensuring better utilization of incident light for photocatalytic reactions on the anode [163]. Hierarchical porous architectures further optimize light absorption through multiple refractions and reflections. Other than that, structural defects within porous materials introduce additional reactive sites and optimize the microstructure, further enhancing the photocatalytic properties. In fact, Yu et al. agreed that surface defects such as steps and kinks in chemically etched porous gold enhance catalytic activity by providing additional reactive sites [41]. Likewise, lattice imperfections in dealloyed CuS improve hydroxyl radical generation, crucial for organic dye degradation for better photocatalysis process [162]. Similarly, the robust architecture of dealloyed porous CuS ensures stability over repeated cycles. Xu et al. reported that CuS catalysts retained over 90% of their efficiency after five photocatalytic cycles, demonstrating excellent reusability [162]. Moreover, beyond these functional benefits, porous materials are versatile and easily modifiable, allowing the incorporation of diverse photoactive materials, making them suitable for various applications [164]. Porous photocatalysts are highly effective in environmental remediation and energy generation due to their ability to adsorb and degrade pollutants efficiently [165].

Table 3 summarizes a concise overview of synthesis methods, morphology and cyclic performance of dealloyed nanoporous anodes. Obviously, variations in dealloying processes produce unique porous structures, each with a distinct impact on electrochemical properties. This summary enables efficient comparison, offering insights into the synthesis techniques and morphological attributes that influence performance outcomes. Undeniably, regardless of the varying pore designs, it consistently shows significant improvements and outperform traditional planar anode materials.

**Table 3.** Synthesis methods, structural characteristics, morphology and cyclic performance of dealloyed nanoporous anodes for energy storage applications.

Synthesis method	Structural characteristic	Morphology	Energy system	Cyclic performance	Ref.
Chemical dealloying of Zn component from Ni-Zn foam in 3 mol/L NaOH	Pores with platelike particle aggregates forming slit-like Ni structures	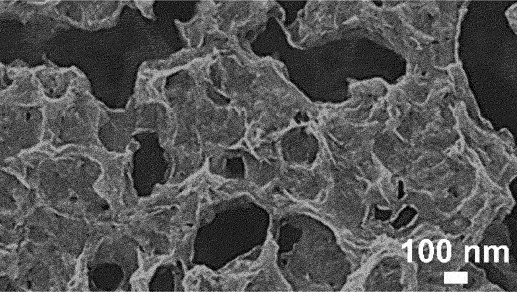	LIBs	Achieved stable voltage profiles at 1 mA/cm^2^ for 1000 h	[137]
Chemical dealloying of Al component from Al-Si-Sn-Sr precursor in 1 M HCl	Multi-dimensional network with abundant voids and secondary dendrites	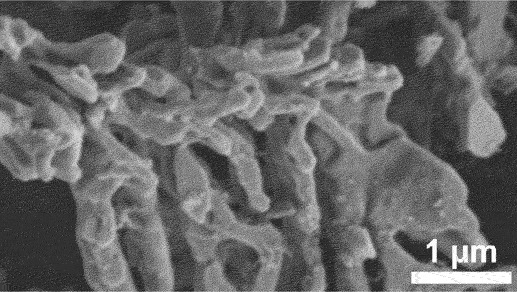	LIBs	Achieved cycle stability with capacity of 935 mAh/g after 1000 cycles	[166]
Chemical etching of Al component from Al-70 wt.% Si precursor in 2 M HCl	Si powder with highly developed porous structure	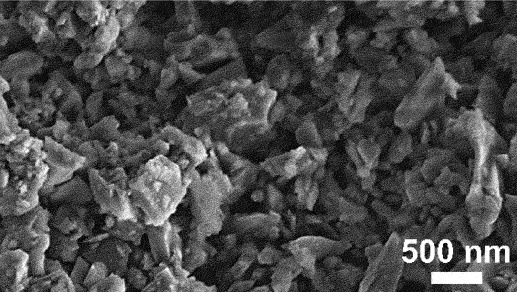	LIBs	Achieved cycle stability with capacity of 1110 mAh/g after 200 cycles	[136]
Chemical dealloying of Al component from Al-Si-Cu powder in 2 M HCl	Continuous porous crystal flower with thin and hollow architecture aligned to pores	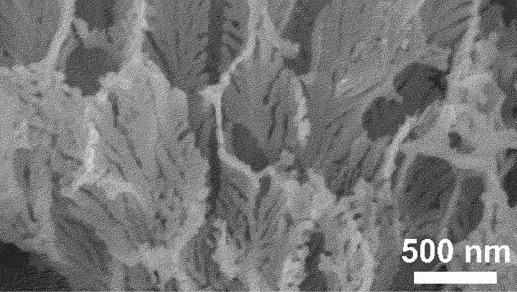	LIBs	Achieved cycle stability with capacity of 914.1 mAh/g after 500 cycles	[167]
Chemical dealloying of Al component from Ge_4.5_Ti_0.5_Al_95_ (at.%) precursor in 0.01 mol/L NaOH	3D pores with interconnected network and aggregated particles anchored along ligaments	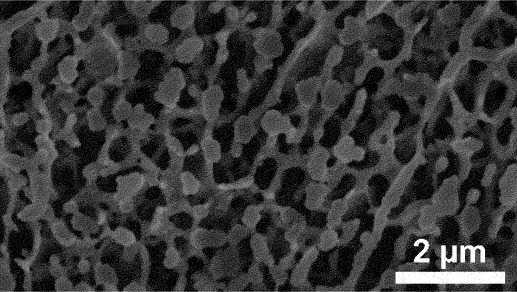	LIBs	Achieved cycle stability with capacity of 963 mAh/g after 100 cycles	[168]
Chemical dealloying of Al component from Zn_50_Al_50_ (at.%) precursor in 1 M KOH	3D nanopores with interpenetrative channels and interconnected Zn ligaments	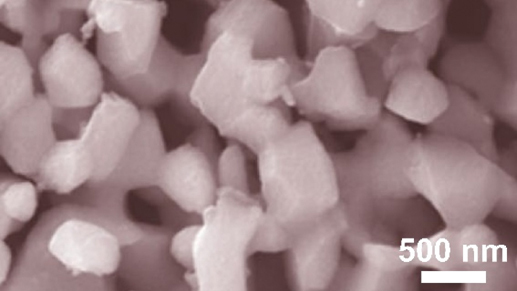	ZIBs	Achieved stable voltage profiles at 0.5 mA/cm^2^ for 1900 h	[138]
Chemical dealloying of Al component from Al_82_Cu_18_ (at.%) precursor in 1 M HCl	3D lamellar porous architecture that consists of quasi-periodic lamellar channels	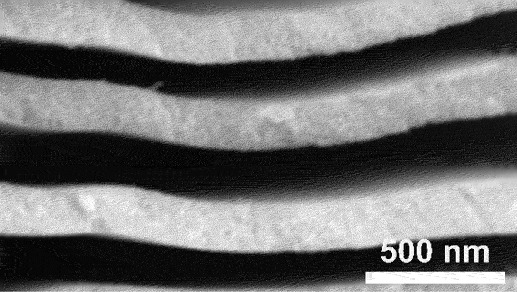	ZIBs	Achieved stable voltage profiles at 0.5 mA/cm^2^ for 4000 h	[77]
Chemical dealloying of Ti_60_Cu_40_ (at.%) precursor alloy strips in 12 M H_2_SO_4_	Porous structure with nano-flowers built by numerous nanosheets	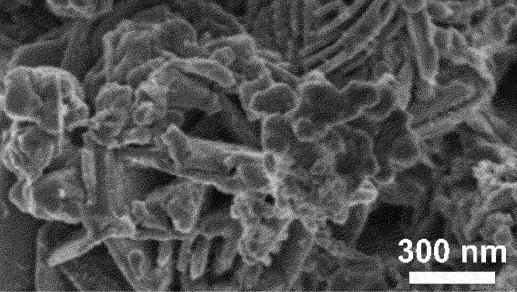	SIBs	Achieved cycle stability with capacity of 154.4 mAh/g after 3000 cycles	[142]
Chemical dealloying of Zn component from Zn-Sb precursor in NaOH	3D mesoporous Sb anode with rough and thin channel	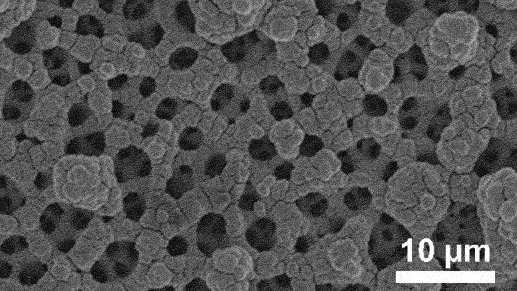	SIBs	Achieved cycle stability with capacity of 575 mAh/g after 200 cycles	[141]
Chemical dealloying of Al component from Al-Ge alloy strips in 5% HCl solution at 60 °C	Slit-type porous structure of germanium anode comprised of thick ligaments	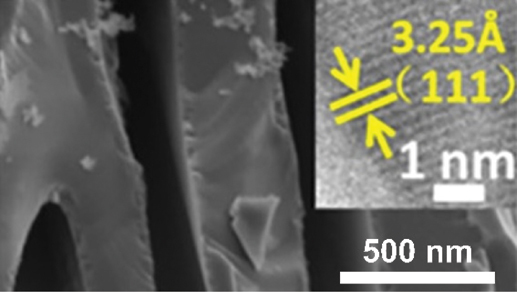	KIBs	Achieved cycle stability with capacity of 120 mAh/g after 400 cycles	[144]
Electrochemical dealloying of Cu component from dendritic Ni – Cu foam in 1 M H_2_SO_4_	Porous Ni – NiO with numerous intertwined nanodendrites	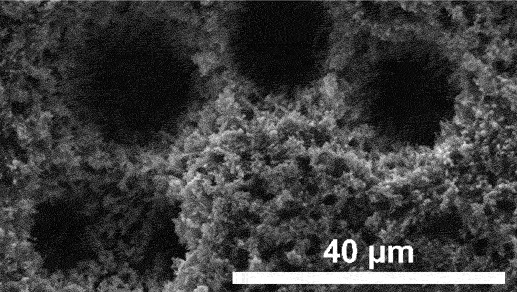	Super-capacitor	Retained 95.1% of the capacitance after 6000 cycles	[153]
Chemical dealloying of Al component from compacted Co Al precursor in 5 M NaOH	Skeletal Co structure decorated with disordered rod-like pores	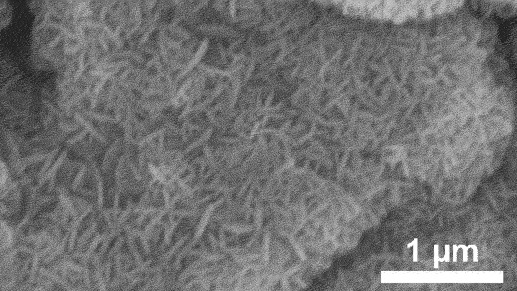	Super-capacitor	Retained 95.83% of the capacitance after 2000 cycles	[169]
Electrochemical dealloying of Cu component from Ni-Cu foam in 0.6 M boric acid	Roughened structured surface decorated with porous Ni nanoparticles	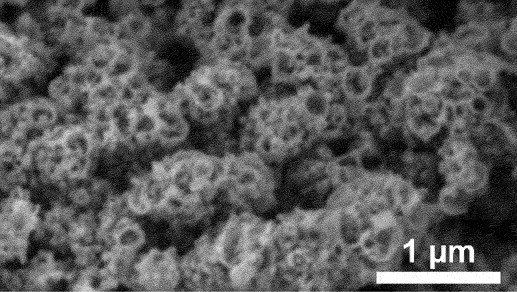	Super-capacitor	Retained 86% of the capacitance after 3000 cycles	[170]
Chemical dealloying of Zr component from Ni_30_Co_10_Zr_60_ (at.%) ribbons in 0.05 M HF	Crack-like pores and corrosive voids within the petal-like microstructure	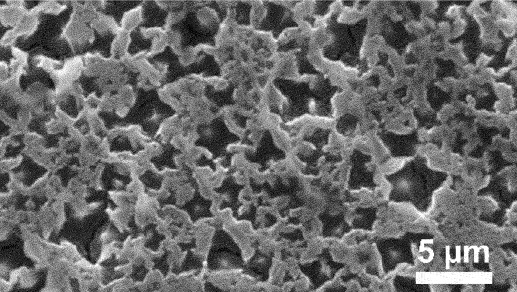	Super-capacitor	Retained 91.3% of the capacitance after 8000 cycles	[154]
Chemical dealloying of Mn component from Cu_30_Mn_70_ (wt.%) strip in 20 vol % acetic acid	Nanoporous MnO_2_ precipitates on Cu-rich substrate	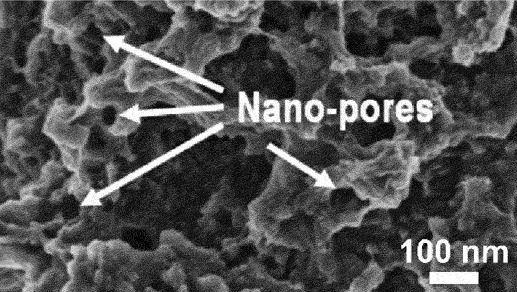	Super-capacitor	Retained 95% of the capacitance after 4000 cycles	[155]
Chemical dealloying of Al component from Ni_10_Al_90_ (at.%) strip in 25 wt% NaOH	Porous Ni with macropore sizes of 100–300 nm and skeleton sizes of 80–200 nm	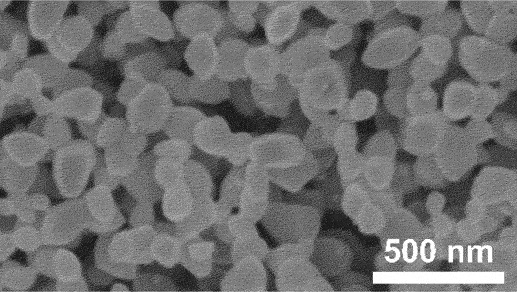	Water splitting	Achieved stability with 22 mV decrease in overpotential after 1000 cycles of cyclic voltammetry	[156]
Chemical dealloying of Al component from Ni_2.5_Co_2.5_Al_95_ strip (at.%) strip in 25 wt% KOH	Ni_2.5_Co_2.5_ alloy with highly developed 3D porous structure	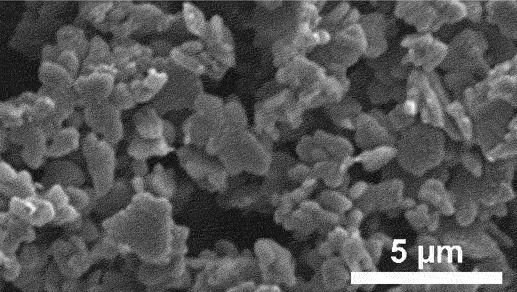	Water splitting	Achieved stability with 7 mV decrease in overpotential after 1000 cycles of cyclic voltammetry	[157]
Chemical dealloying of Mg component from Cu_28_Mg_72_ ribbon in 0.68 M HCl	Interconnected pore channels and Cu ligaments with size of 100 ~ 200 nm	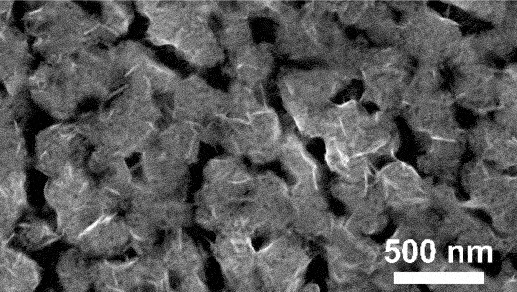	Water splitting	Achieved durability with overpotential of 310 mV over 10 h	[158]
Chemical dealloying of Ti component from Ti-Cu precursor in H_2_SO_4_	Nanoporous structure of CuS with interconnected channels and ligaments	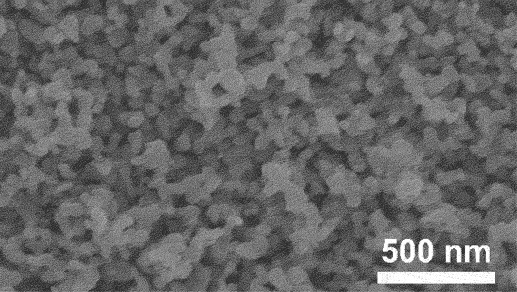	Photo-catalyst	Retained 90% efficiency after 5 photocatalytic cycles	[162]

## Conclusion

Nanoporous materials can be effectively synthesized through facile dealloying techniques. The distinctive structural and functional properties make them indispensable for advancing modern technologies. In this review, we focus on nanoporous anode fabricated by chemical and electrochemical dealloying, specifically for energy storage applications like LIBs, ZIBs, SIBs, KIBs, supercapacitors and anodic OER of water splitting and photocatalyst. Porous design has interconnected open pores with extensive surface area that provide abundant channels for charge transfer. The highly adjustable pore sizes ranging from the nanoscale grade to microscale grade enhance electrolyte penetration and ion diffusion throughout the anode. This structural flexibility improves electrochemical performance and allows the anode to accommodate significant volume changes during charge-discharge cycle which maintains the cycle stability. With ongoing advancements in the dealloying technique, researchers continue to explore its potential for producing nanomaterials tailored to energy storage needs. Developing low-cost, scalable, straightforward and controllable dealloying methods could be a major driver for the commercialization of nanoporous anodes in the near future.

However, very high volume fraction of the pore phase can lead to excessive electrolyte consumption, irreversible capacity loss and low utilization of active materials. Consequently, energy storage systems may suffer from low volumetric energy density which complicate the integration into compact electronic devices. The intricate relationship between alloy designs and dealloying kinetics presents significant challenges in fabricating well-defined nanoporous structures. While current literature provides a solid foundation, previous work has largely overlooked the collective effects of factors such as temperature, etchant concentration, type of parent alloy, composition, phase distribution, applied potential and dealloying time in fine-tuning porosity for specific designs. Fundamental research has predominantly focused on nanoporous Au derived from binary isomorphous Ag – Au alloy systems. Nevertheless, due to differences in epitaxial nature observed in systems such as metallic glasses and intermetallic compounds, the majority of dealloying systems are not as ideal as this prototypical nanoporous Au. Additionally, high-entropy amorphous alloys remain relatively unexplored, despite their extraordinary properties that could facilitate the development of superior porous structures.

The increased surface area associated with nanoporous materials can induce unwanted side reactions and accelerate capacity degradation over extended cycling times. Although depositing active materials onto nanoporous metals has shown substantial performance improvements, this approach often results in reduced overall energy density due to the added weight of heavy metals and inert components. Given the scarcity of research on lighter alternatives, identifying such materials for energy storage applications should be a future research priority. Furthermore, sequential dealloying offers potential to create hierarchical pore structures with complex configurations which can enhance mass transfer and electrolyte percolation. Despite progress in this area, another key question remains unanswered. Optimizing porosity while balancing mechanical strength and electrochemical performance is still ambiguous. Mechanical properties are essential to ensure the durability and reliability of electrodes. In advanced applications requiring flexibility such as membrane-integrated electronics, medical devices and healthcare monitoring systems, anode materials must maintain stable performance under various mechanical stresses, including bending and twisting. The applications of dealloyed nanoporous materials extend far beyond energy storage, encompassing fields like sensors, actuators, bio-devices and filtration. Currently, most research on nanoporous materials focuses on anodes. However, future efforts should aim to replicate the advances achieved in anodes across other components including cathodes, separators and current collectors.
